# *In-silico study* of seaweed secondary metabolites as AXL kinase inhibitors

**DOI:** 10.1016/j.sjbs.2021.11.054

**Published:** 2021-11-30

**Authors:** Lavanya Nagamalla, J.V. Shanmukha Kumar, Chintakindi Sanjay, Ali M Alsamhan, Mohammed Rafi Shaik

**Affiliations:** a Department of Chemistry, Koneru Lakshmaiah Education Foundation, Vaddeswaram, A.P., India; b Industrial Engineering Department, College of Engineering, King Saud University, P.O. Box. 800, Riyadh 11451, Saudi Arabia; c Department of Chemistry, College of Science, King Saud University, P.O. Box 2455, Riyadh 11451, Saudi Arabia

**Keywords:** AXL kinase, Marine species, Virtual screening, Cancer therapy, Secondary metabolites, Molecular dynamics simulations

## Abstract

AXL kinase is an attractive cancer target for drug design and it is involved in different cancers. A set of molecule databases with 1072 secondary metabolites from seaweeds were screened against the AXL kinase active site and eight molecules were shortlisted for further studies. From the docking analysis of the complexes, four molecules GA011, BE005, BC010, and BC005 are showing prominent binging. From the 100 ns of molecular dynamics simulations and ligand-bound complex MM-PBSA free energy analysis, two molecules BC010 (ΔG = −135.38 kJ/mol) and BE005 (ΔG = −141.72 kJ/mol) are showing molecule stability in the active site also showing very strong binding free energies. It suggests these molecules could be the potent molecules for AXL kinase.

## 1 Introduction

Universally cancer causes millions of deaths every year and nearly half of the newly diagnosed cancer patients can be cured by using present available treatment options (Arends, 2010). The present cancer therapies such as chemotherapy and surgery or a combination of both are rapidly losing efficacy. Present treatment methods are with low cure rate and at the same time, they cause severe adverse effects, which increase the mortality rate of cancer patients. This situation demands the necessity of innovative cancer therapies. The tyrosine kinase AXL pertains TAM family (Tyro3, AXL, Mer) (Brenig et al., 2020, Corno et al., 2016, Liu et al., 1988). AXL activates different signalling pathways by binding with endogenous ligand growth arrest-specific 6 (Gas6) which mediate essential cellular processes namely cell growth, cell survival, differentiation, proliferation, cell adhesion, cell migration, invasion, and angiogenesis, etc (Varnum et al., 1995). The overexpression of AXL kinase is noticed in a great variety of tumors, such as breast cancer (Holland et al., 2010, Zhang et al., 2008), leukemia (Ben-Batalla et al., 2013, Wu et al., 2014), pancreatic cancer (D’Errico et al., 2019, Koorstra et al., 2009), lung cancer (Levin et al., 2016), and in many cases, the over expression correlates with a lower patient prediction. A previous report reveals that when AXL inhibitors are used to treat the resistant cell lines, the sensitivity of the cells is restored to certain drugs (Rho et al., 2014). Consequently, AXL emerges as a novel optimistic molecular target for designing potential anti-cancer drugs.

Natural products and their derivatives are significant sources for drugs. In cancer treatment, it is eminent that natural products are potential drugs (Ruiz-Torres et al., 2017, Seca and Pinto, 2018b). At present not less than 30% of the present best twenty drugs appear from plants and marine species. Now a day, there are 175 approved small molecules in the market to treat cancer; out of them, about 49% of drugs are from natural products (Newman and Cragg, 2016). Investigators concentrating on the preparation of macroalgae extracts and their characterizations disclosed that a large number of seaweed compounds with very significant biological properties comprising antimicrobial, antihypertensive, anti-inflammatory, anticancer activity etc., Macroalgae are progressively observed as a source of secondary metabolites with abundant prospective for the advancement of new drugs. Seaweeds, in accumulation to their usage as food, are present consistently accredited as a vital source of new natural products that might embrace significant principals for upcoming drug discovery and advancement, comprising in the hindrance of the treatment in cardiovascular and anticancer. In this advancement, in vitro studies are only the initial phase in an extensive procedure, whereas in vivo investigations and clinical trials are the utmost illuminating phases of the factual prospective and boundaries that a secondary metabolite might have castoff as a new drug (Hussain et al., 2016, Rocha et al., 2018, Rosa et al., 2020, Seca and Pinto, 2018a, Veríssimo et al., 2021). Seaweeds show a significant part in ocean biodiversity and contain green, brown, and red algae. Secondary metabolites of seaweeds show promising bioactivities (El Gamal, 2010, Elansary et al., 2016, Pérez et al., 2016, Rosa et al., 2020, Seca and Pinto, 2018a, Sithranga Boopathy and Kathiresan, 2010, Wan-Loy and Siew-Moi, 2016). In recent years, researchers found that seaweed metabolites are plentiful of terpenoids, alkaloids, polyphenols, steroids, pigments, and polysaccharides. The assessment of pharmacological activities of these metabolites exhibited efficient biological activities (Cardoso et al., 2015, Gouveia et al., 2013, Sun et al., 2018, Tiwari and Troy, 2015) preferably showed anti-cancer properties (Folmer et al., 2010, Murphy et al., 2014).

**Scheme 1:**
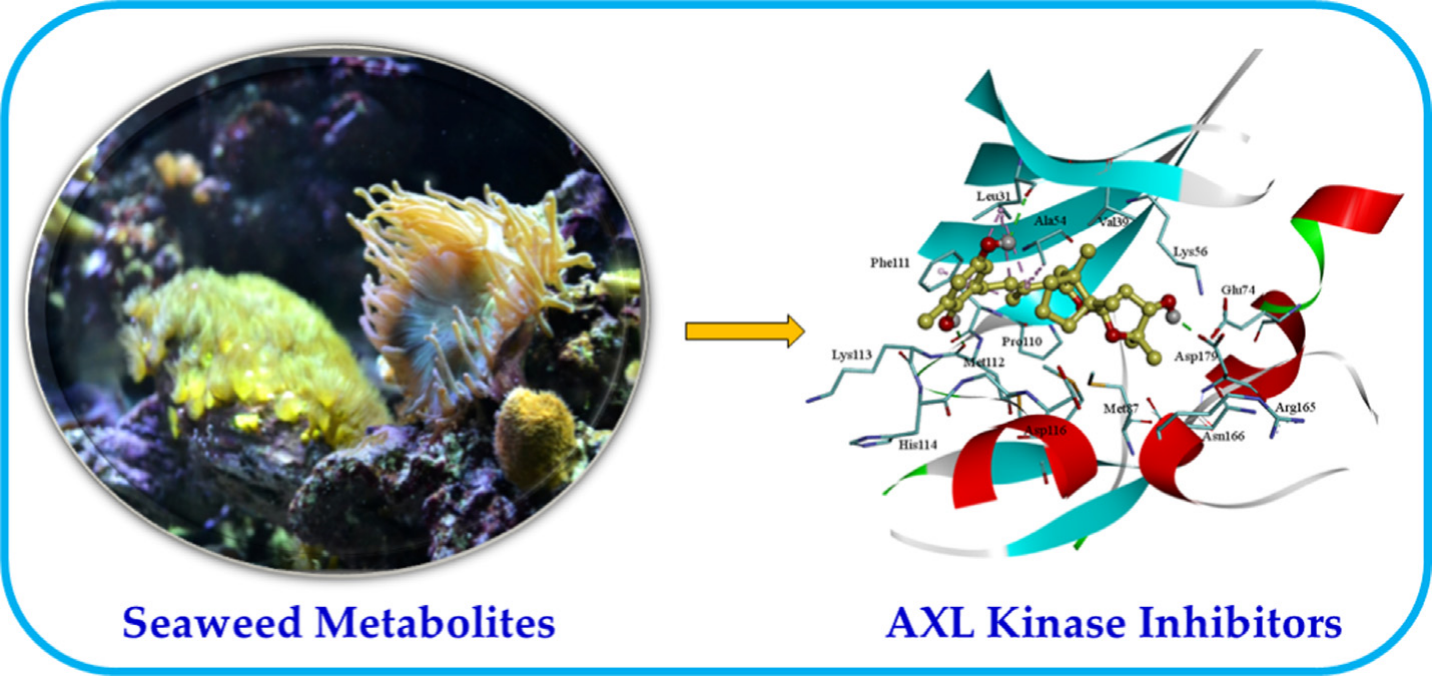
Graphical representation of AXL inhibitor activity of secondary metabolites from seaweed inhibitors.

In the present in-silico study, virtual screening is performed on AXL protein target with open-access database (Davis and Vasanthi, 2011) of secondary metabolites from seaweeds (1072 molecules). Furthermore, the docking studies of molecular were conducted on active molecules. To understand the protein–ligand interactions the four most active complexes were chosen for free energy and molecular dynamics investigations. This in-silico study aimed to understand the AXL inhibitor activity of secondary metabolites from seaweed inhibitors (Scheme 1).

## 2 Methodology

### 2.1 Virtual screening

A data set of 1072 molecules from an open-access database of secondary metabolites from seaweeds (https://www.swmd.co.in/) was prepared for the virtual screening (Davis and Vasanthi, 2011). The virtual screening approach is a helpful tool in detecting potential principles from vast chemical databases in contrast to specific target proteins (Gaur et al., 2018). The coordinates of target AXL kinase protein and the molecules from the database were converted into autodock suitable .pdbqt format by using AutodockTools software (Morris et al., 2009). Autodock Vina is used to perform virtual screening of the database against AXL kinase (Trott and Olson, 2010). The AXL kinase domain crystal structure (PDB_ID: 5U6B) was taken from the RCSB protein databank. The protein missing residues were built by SWISS-MODEL online tools with the same crystal template. The Auto ligand preparation tool was castoff to comprise Gasteiger partial charges and polar hydrogen atoms to each ligand in the database. AutodockTools software was castoff to set up a grid around the AXL protein binding site and the center of the grid −20.518 × −4.68 × 10.651 and the grid dimensions were fixed as 20 × 18 × 34 Å^3^ with points divided by 1.0 Å. Upon completion of docking, the generated docking conformers were visualized and analyzed using pymol software.

### 2.2 Molecular dynamics and free energy calculations

The selected five complexes protein–ligand were subjected to 100 ns (nanoseconds) simulations of molecular dynamics (MD) under constant pressure and temperature conditions and the stability of the molecules can be understood with simulations of MD complexes (Sakthivel and Habeeb, 2018, Tanneeru et al., 2015, Tanneeru and Guruprasad, 2013, Wan et al., 2019). All the simulations of MD were conducted by using Gromacs 2018, Ubuntu-18.04 LTS Linux platform. The protein force fields were assigned by using the GROMOS96 53a6 force field and the inhibitor molecules force fields were generated by PRODRG online web server (Oostenbrink et al., 2004). A box cubic near the complexes of protein–ligand is made with a 10 Å edge length, and the box cubic is packed with SPC water models as solvent molecules. The complex system was added with Na^+^ ions to neutralize negative charges. Energy minimization was performed using the sharpest descent depreciation method on the protein–ligand complex system and to adjust the water molecules around the system a 100 pico seconds (ps) of position restrained molecular dynamics were conducted. A total of MD simulations 100 ns of were achieved on the complex system using 0.002 ps time step. An isothermal and isobaric ensemble was applied to the system and the V-rescale method thermostat is used to stabilize the temperature of the total system (Bussi et al., 2007). One bar pressure was created by applying the Parrinello Rahman approach (Parrinello and Rahman, 1981). The algorithm LINCS is applied to compel the hydrogen (H_2_) bonds (Hess et al., 1997). The system coordinates path files are collected from the total 100 ns of simulations and the coordinates are used to calculate Molecular Mechanics - Poisson Boltzmann Surface Area (MM-PBSA) free energies. The mm_pbsa is used to calculate the binding ligand-free energy with kinase protein AXL (Kumari et al., 2014).

The ligand binding to the protein active site free energy change in terms of ΔG_binding_ is calculated with the following equation:

$$\Delta G_{\mathrm{binding}}=G_{\left(protein+ligand\right)}-\left(G_{\mathrm{protein}}+G_{\mathrm{ligand}}\right)$$

where *G_protein+ligand_* is the total protein–ligand complex free energy and, *G_protein_* and *G_ligand_* are individual free energies calculated in a solvent,

$$G=\langle E_{\mathrm{MM}}\rangle -TS+\langle G_{\mathrm{solvation}}\rangle$$

where the average molecular mechanic's vacuum potential energy is 〈*E*_MM_〉. The T is temperature, S is the entropic contribution, respectively, and the 〈*G_solvation_*〉 is the free energy of salvation of each component. The free energy (G) can be calculated for all individual components for the protein, ligand, and protein–ligand complex.

$$E_{\mathrm{MM}}=E_{\mathrm{bonded}}+E_{\mathrm{non}-bonded}=E_{\mathrm{bonded}}+\;\left(E_{\mathrm{vdW}}+E_{\mathrm{elec}}\right)$$

where bonded interaction is *E_bonded_* that including of dihedral, bond, angle, and inappropriate interactions energies. The *E_non-bonded_* is non-bonded interactions is a combination of both interactions energies electrostatic (*E_elec_*) and van der Waals (*E_vdW_*).

The required energy to transference a solute from a solvent vacuum is the free energy of salvations and it is the combination of the G*_polar,_* an electrostatic and G*_non-polar_* a non-electrostatic element.

$$G_{\mathrm{solvation}}=G_{\mathrm{polar}}+G_{\mathrm{non}-polar}$$

The polar solvation free energy can be is estimated by solving the MM-PBSA equation and the non-polar free energy is calculated from the Solvent existing surface area (SASA) non-polar model.

## 3 Results

### 3.1 Virtual screening analysis

Seaweed Metabolite Database comprises molecules mostly from the Red algae *Laurencia* spp. Virtual screening of molecules 1072 using Autodock Vina tool on kinase AXL active site was performed to get best-hit conformations. Among the 1072 molecules screening top 100 hits were taken as cut-off of compounds with high binding affinities and further evolution of these top 100 molecules was carried out by structural visualization. The various non-covalent interactions play a significant part in the constancy of these protein–ligand complex stability (Purushotham and Sastry, 2014, Purushotham et al., 2012, Uppula, 2018). Depending on the molecular docking conformation orientation in the site active further 100 molecules were reduced to the top 10 molecules.

The binding interactions of the top 8 molecules BC005, BC010, BE005, BE012, BE015, GA011, GA012, and RL516 are indicating that these molecules are stabilized with good binding affinities. The active site of the AXL kinase comprises Leu31, Glu35, Val39, Ala54, Lys56, Glu74, Met87, Pro110, Phe111, Met112, Lys113, His114, Asp116, Ser119, Tyr123, Arg165, Asn166, Asp179, Met200, and Pro201 residues.

#### 3.1.1 BC005

**Fig. 1:**
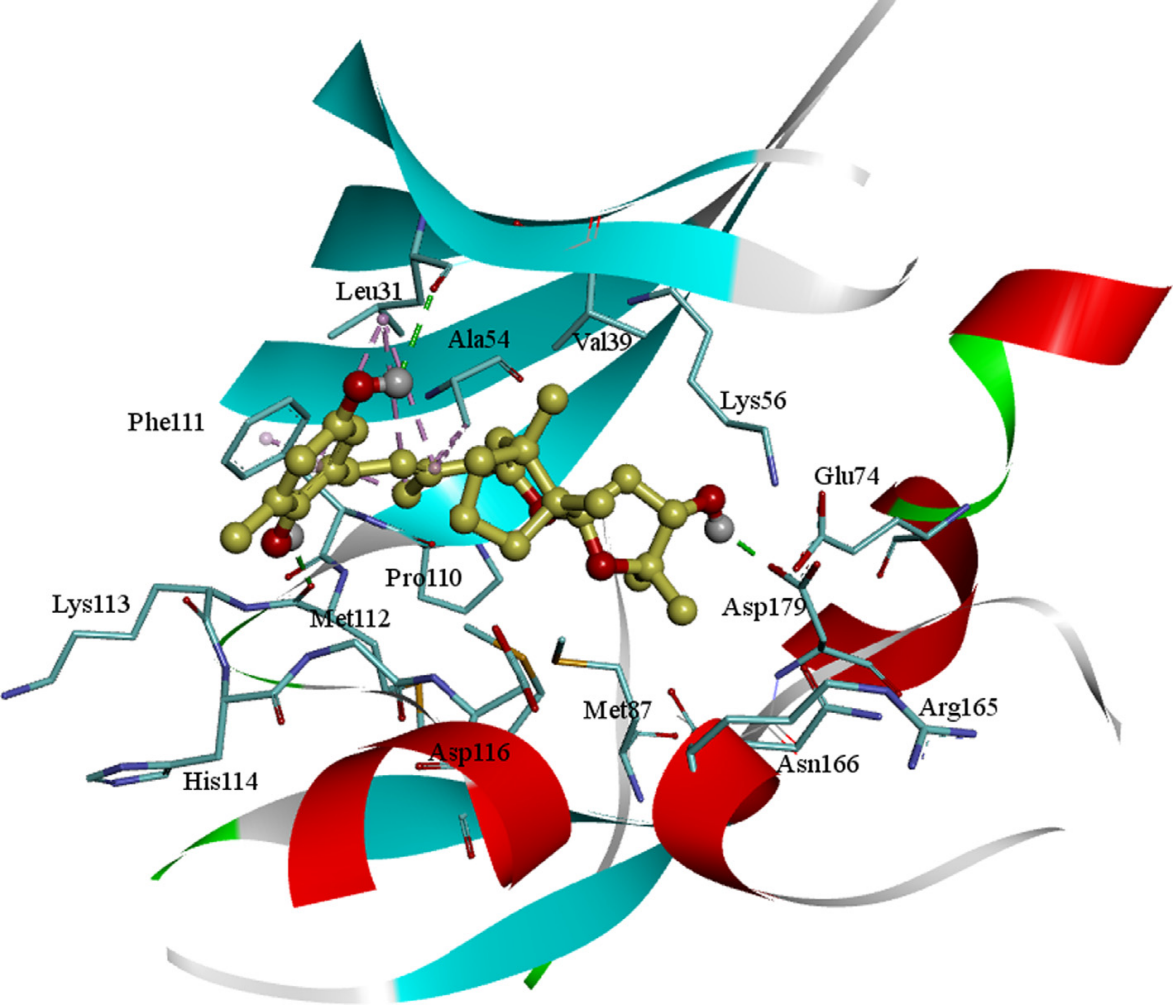
BC005 Molecular docking into the AXL kinase active site. The residue's active site is exhibited in blue sticks and flat ribbon style. Inhibitor molecules are revealed in yellow sticks. All the Hydrogen (H_2_) bonds are exhibited in green broken lines.

The docked molecule BC005 into the active site of the AXL kinase is stabilized by nonbonded interactions like hydrophobic and hydrogen bonds. The docking conformation of the BC005 molecule is shown in Fig. 1. The *ortho*- hydroxyl –OH group of the phenyl ring is establishing an H_2_ bond with the principle-chain carbonyl oxygen of Met112 residue. The *para*-hydroxyl –OH group of the phenyl ring is establishing an H_2_ bond with the main-chain carbonyl oxygen of Leu31 residue. The –OH group on the 2′-oxolane rings of the spiro-group formed an H_2_ bond with the adjacent chain carbonyl of Asp179. This molecule shows a CH-π interaction with the Phe111 aromatic ring and other active site residues hydrophobic interactions.

#### 3.1.2 BC010

**Fig. 2:**
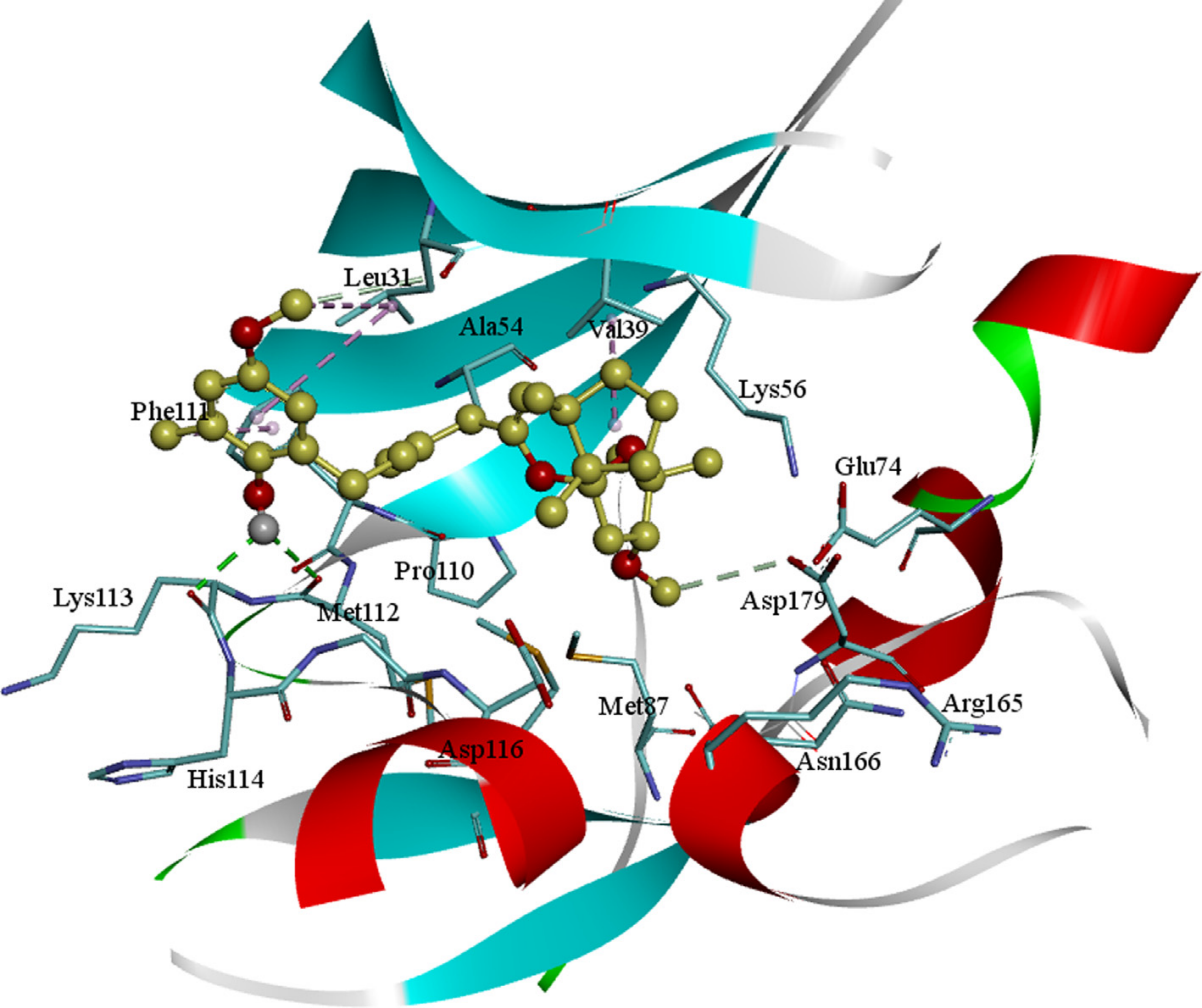
BC010Molecular docking of AXL kinase active site. The residue's active site is exhibited in blue sticks and flat ribbon style. Inhibitor molecules are exhibited in yellow sticks. All the H_2_ bonds are shown in green broken lines.

The molecule BC010 is forming hydrogen bonds, hydrophobic and non-bonded contacts with the active site amino acids of AXL kinase. The *ortho*- hydroxyl group –OH of the phenyl ring is creating a characteristic H_2_ bond with the hinge region residues and it is developing a bifurcated hydrogen bond with the principal chain of Met112 residue carbonyl oxygen and the main-chain carbonyl oxygen of Lys113 residues. The side chain of Leu31 is forming a CH-π interaction with the BC010 aromatic ring of the molecule. The spiro ring is stabilized by hydrophobic residues such as Val39, Ala54, Lys56, and Met87 residues (Fig. 2).

#### 3.1.3 BE005

**Fig. 3:**
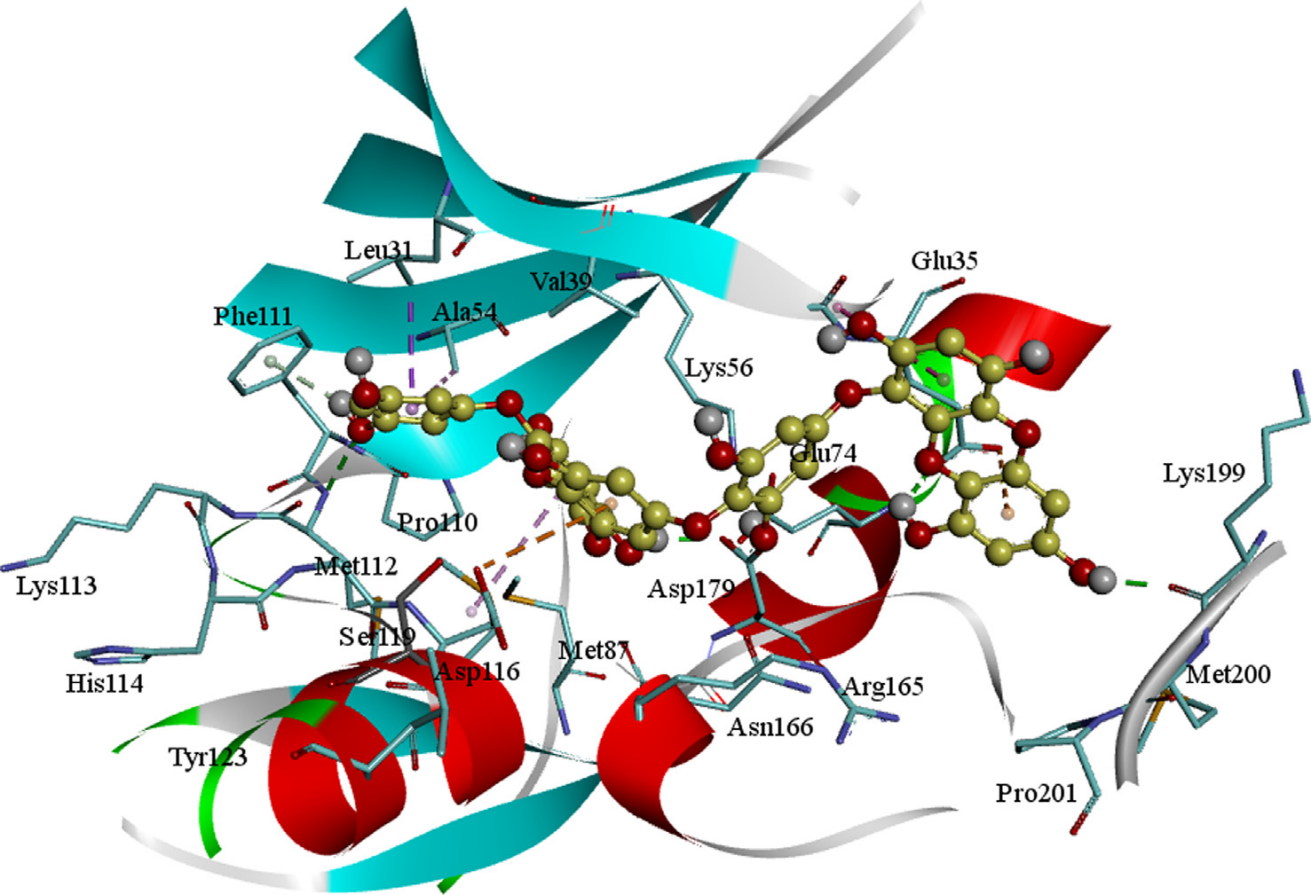
BE005Molecular docking into the AXL kinase active site. The residues active site is exhibited in blue sticks and flat ribbon style. Inhibitor molecules are exhibited in yellow color sticks. All the H_2_ bonds are exhibited in green color broken lines.

The molecule BE005 is stabilized in the AXL kinase active site and a characteristic H_2_ bond was noticed among the hydroxyl oxygen of the ligand molecule with the foremost chain NH of Met112 residue. A hydroxyl –OH of ligand molecule is forming an H_2_ bond with adjacent-chain Glu35 carbonyl oxygen amino acid and other hydroxyls on oxanthrene ring are showed a hydrogen bond with the principal chain NH of Lys199 residue (Fig. 3). The side-chain carboxylic acid group of Asp116 residue is establishing an anion-π interaction with one of the aromatic rings of the ligand. In the similar, the Glu35 side chain also forms an anion-π interaction with the aromatic ring of oxanthrene. The side chain of Leu31 residue is forming a CH-π interaction with a ligand molecule aromatic ring.

#### 3.1.4 BE012

**Fig. 4:**
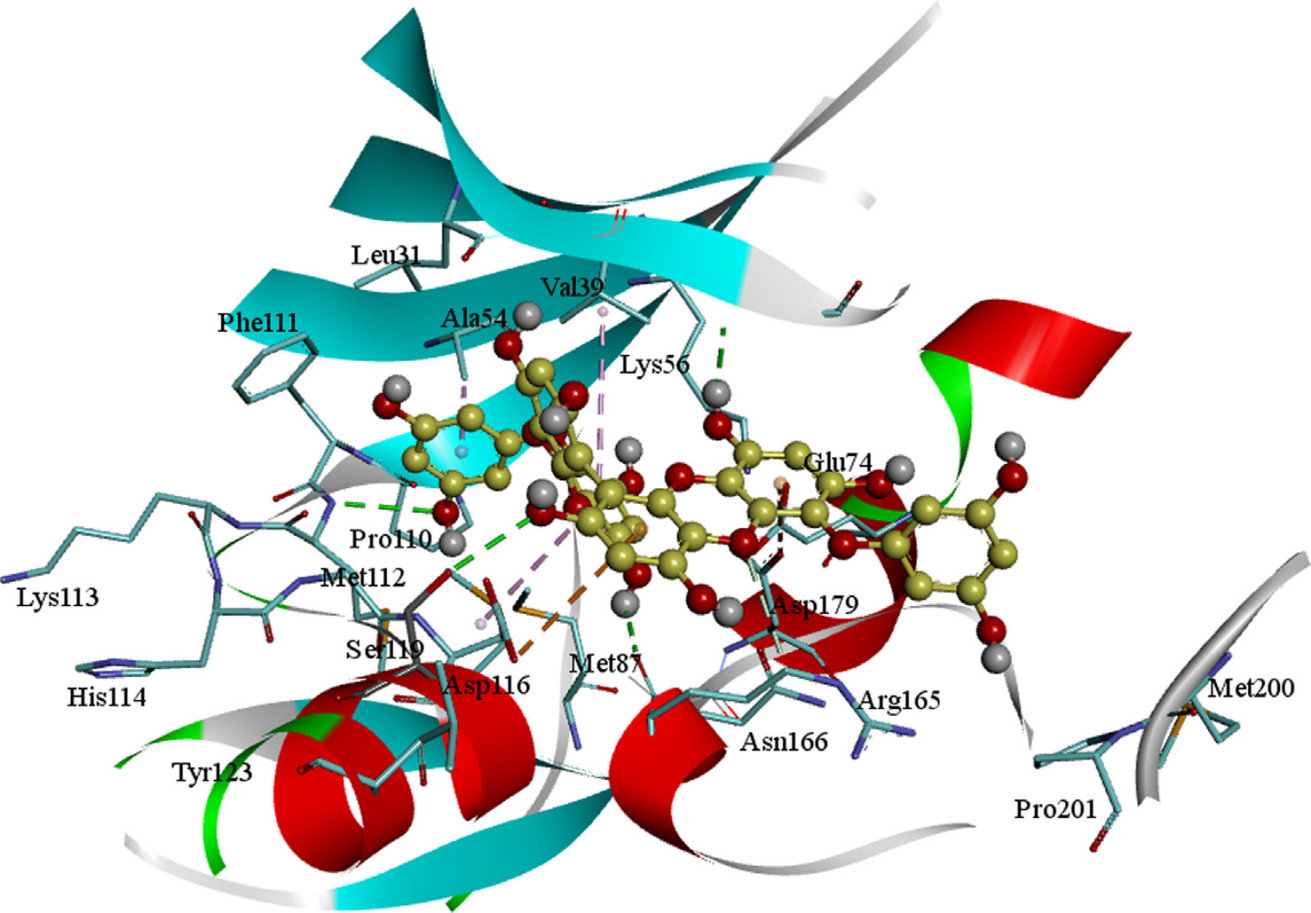
Molecular docking of BE012 into the AXL kinase active site. The residues active site is exhibited in blue sticks and flat ribbon style. Inhibitor molecules are exhibited in yellow color sticks. All the H_2_ bonds are exhibited in green color broken lines.

The molecule BE012 is stabilized in the active site of AXL kinase. The Hydroxyl oxygen is developing an H_2_ bond with the principal chain NH of Met112 on the hinge section. The hydroxyl OH group on the oxanthrene ring is creating a hydrogen bond with the side-chain oxygen of Ser119 residue and one more hydroxyl OH group on another oxanthrene ring is forming a hydrogen bond with the main-chain carbonyl oxygen of Arg165 residue. One of the di hydroxyl phenyl ring is stabilized by CH-π interactions with the side chain of Ala54 residue. An anion-π interaction is observed among the aromatic ring and the carbonyl group of Asp179 residue (Fig. 4). The molecule is also steadied by other active site hydrophobic interactions.

#### 3.1.5 BE015

**Fig. 5:**
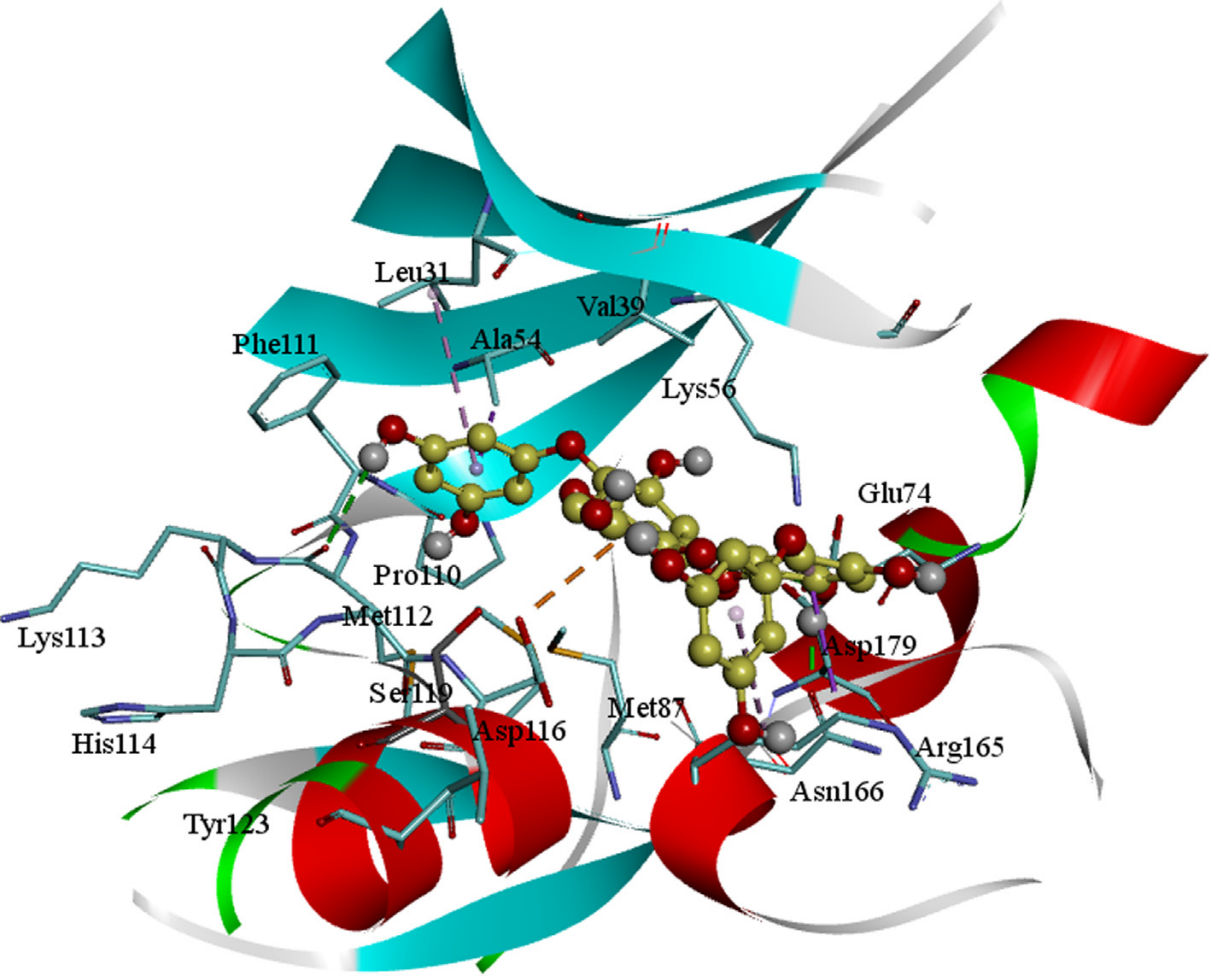
Molecular docking of BE015 into the active site of AXL kinase. Active site residues are shown in blue sticks and flat ribbon style. Inhibitor molecules are exhibited in yellow color sticks. Hydrogen bonds all are shown in green color broken lines.

The molecule BE015 hydroxyl OH is establishing an H_2_ bond with the principal chain carbonyl of Met112 at the hinge region of AXL kinase. Hydroxyl OH group on benzofuro ring is developing a hydrogen bond with the adjacent chain carbonyl oxygen of Asp179 residue and the hydroxyl OH group on oxanthrene ring is establishing a hydrogen bond with the side-chain oxygen of Asn166 residue. The di hydroxyl phenyl ring is stabilized by CH-π interactions with the adjacent chain of Ala54 residue and Leu31 residues (Fig. 5).

#### 3.1.6 GA011

**Fig. 6:**
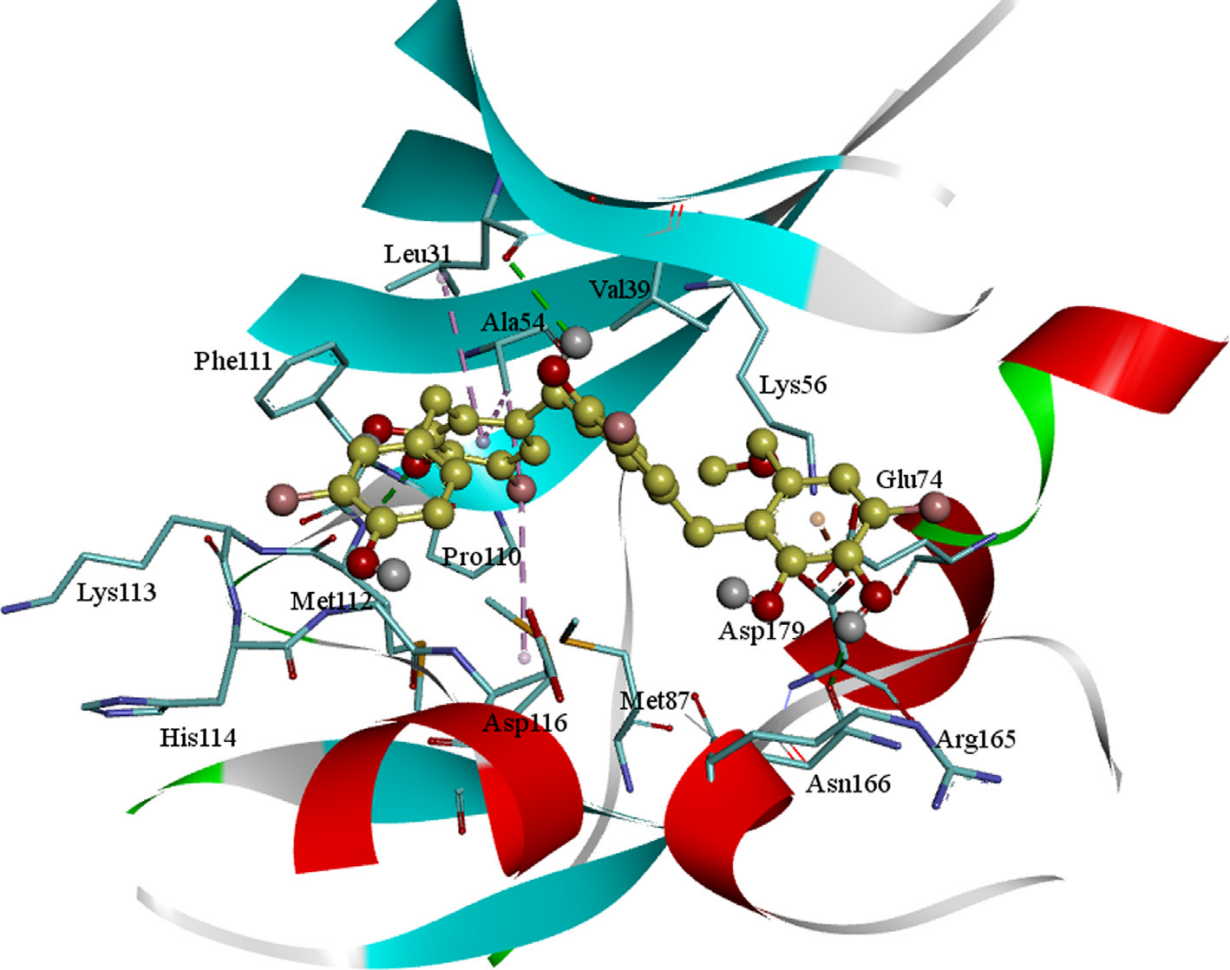
Molecular docking of GA011 into the active site of the AXL kinase. Active site residues are shown in blue sticks and flat ribbon style. Inhibitor molecules are exhibited in yellow color sticks. Hydrogen bonds all are shown in green color broken lines.

The molecule GA011 is stabilized by hydrogen bonds formed with the active site of the AXL kinase. The hydroxyl OH of the bromo di-hydroxy benzyl ring is developing a hydrogen bond with principal-chain NH of Met112 amino acid residue on the hinge region and the aromatic ring is stabilized by CH-π interactions with the side chain of Ala54 residue and Leu31 residues. At the middle of the molecule, the OH group of bromo hydroxybenzyl is establishing a hydrogen bond with the main-chain carbonyl oxygen of Leu31 residue and its ring is forming an anion-π interaction with the adjacent chain carbonyl group of Asp116. At the terminal of a molecule, the OH group of bromo di-hydroxybenzyl is creating a hydrogen bond with the side-chain carbonyl oxygen of Asn166 residue and its ring is forming an interaction anion-π with the adjacent chain of Asp179 carbonyl group (Fig. 6).

#### 3.1.7 GA012

**Fig. 7:**
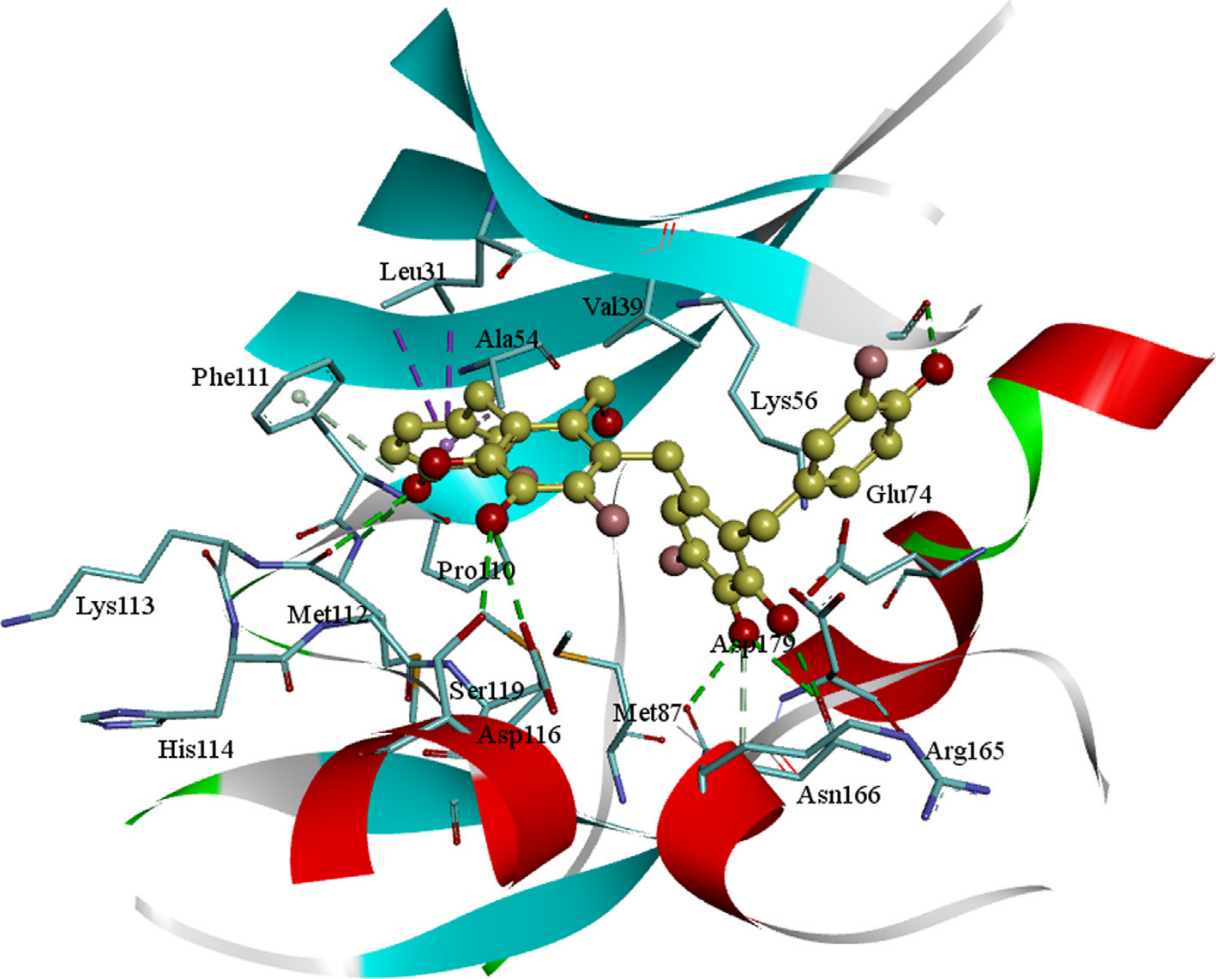
Molecular docking of GA012 into the active site of the AXL kinase. Active site residues are shown in blue sticks and flat ribbon style. Inhibitor molecules are exhibited in yellow color sticks. Hydrogen bonds all are shown in green color broken lines.

The GA012 molecule is forming a characteristic H_2_ bond at the hinge region with the main chain NH of Met112 residue and the aromatic ring is stabilized by CH-π interactions with the adjacent chain of Ala54 residue and Leu31 residues (Fig. 7). The other hydroxyl group of molecules is forming separated hydrogen bonds with adjacent chains of Asp116 and Ser119 residues. One more hydroxyl OH is forming bifurcated hydrogen bonds with adjacent chain carbonyl of Asn166 and main-chain carbonyl of Arg165 residues.

#### 3.1.8 RL516

**Fig. 8:**
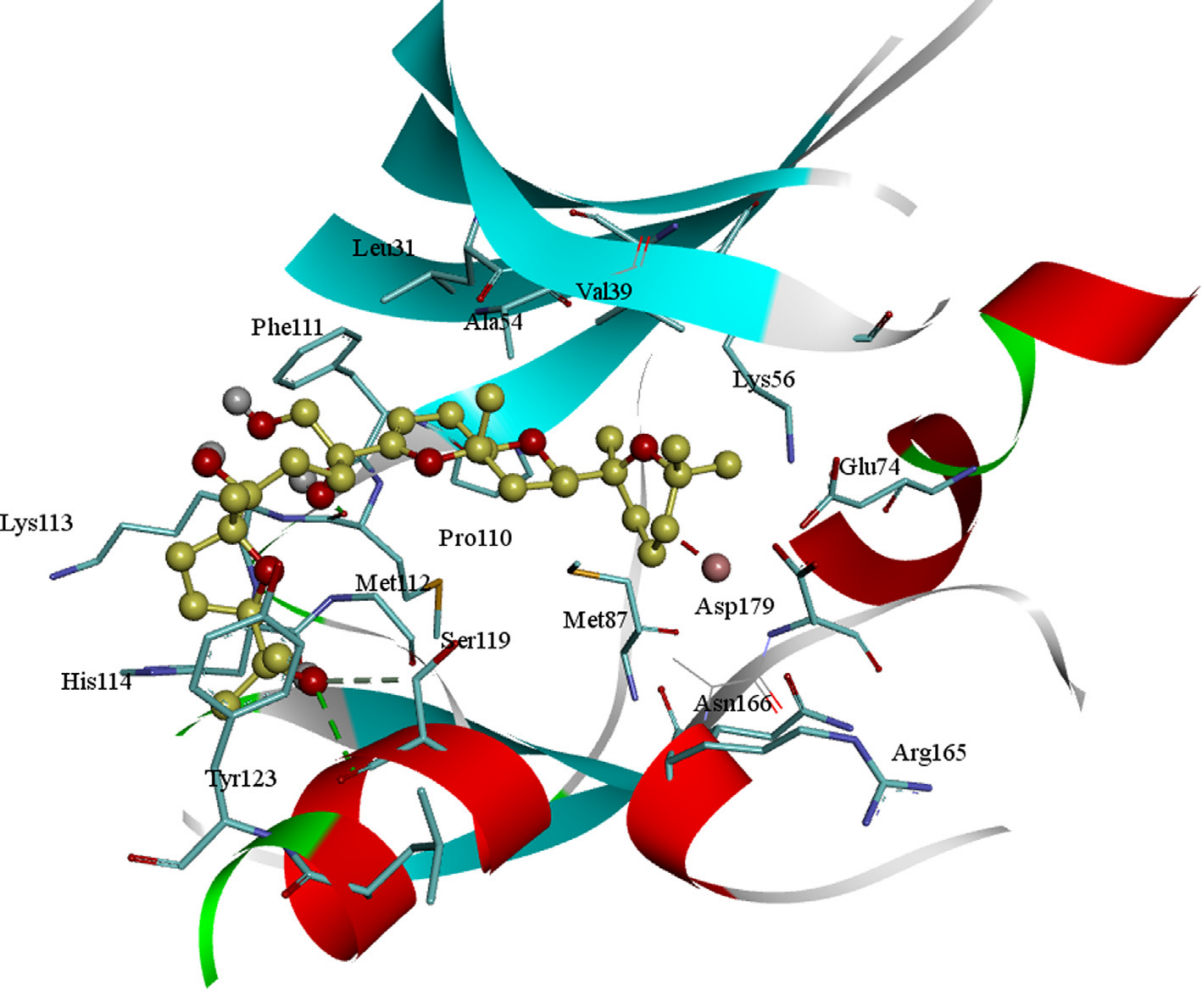
RL516 molecular docking into the active site of the AXL kinase. Active site residues are shown in blue sticks and flat ribbon style. Inhibitor molecules are exhibited in yellow sticks. The hydrogen bonds all are shown in green color broken lines.

The RL516 molecule hydroxyl OH is developing a characteristic H_2_ bond at the hinge region with principal-chain NH of Met112 residue. The OH group of 2-hydroxy-2-propanyl is forming a divided hydrogen bond with the principal-chain carbonyl of His114 residue and main-chain NH of Ser119 residues (Fig. 8). The molecule is stabilized by hydrophobic interactions in the active site.

## 4 Discussions

### 4.1 Molecular dynamics simulations

**Fig. 9:**
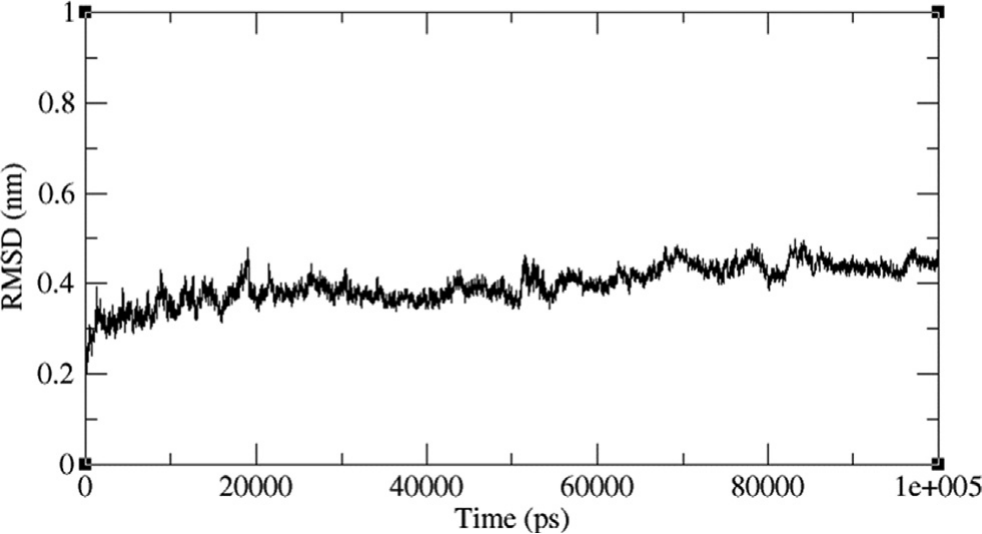
RMSD plot of AXL kinase protein in complex with BC005 molecule.

**Fig. 10:**
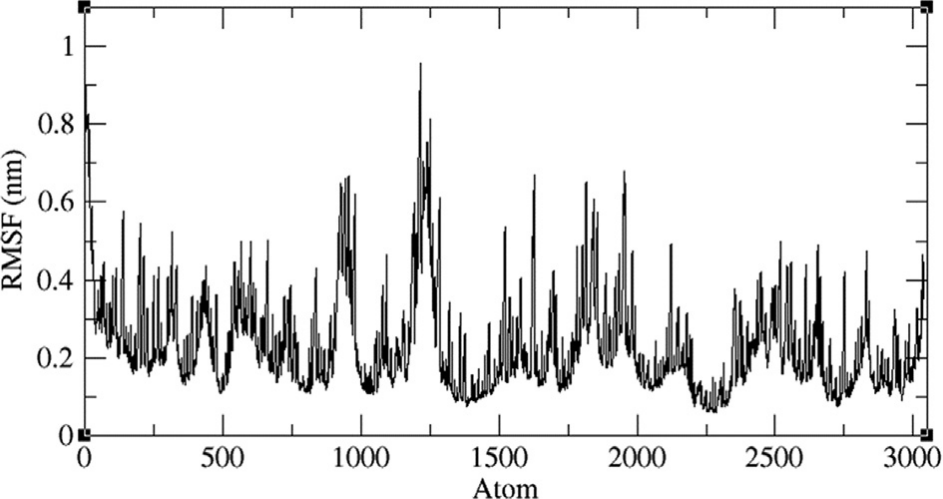
RMSF plot of AXL kinase protein in complex with BC005 molecule.

**Fig. 11:**
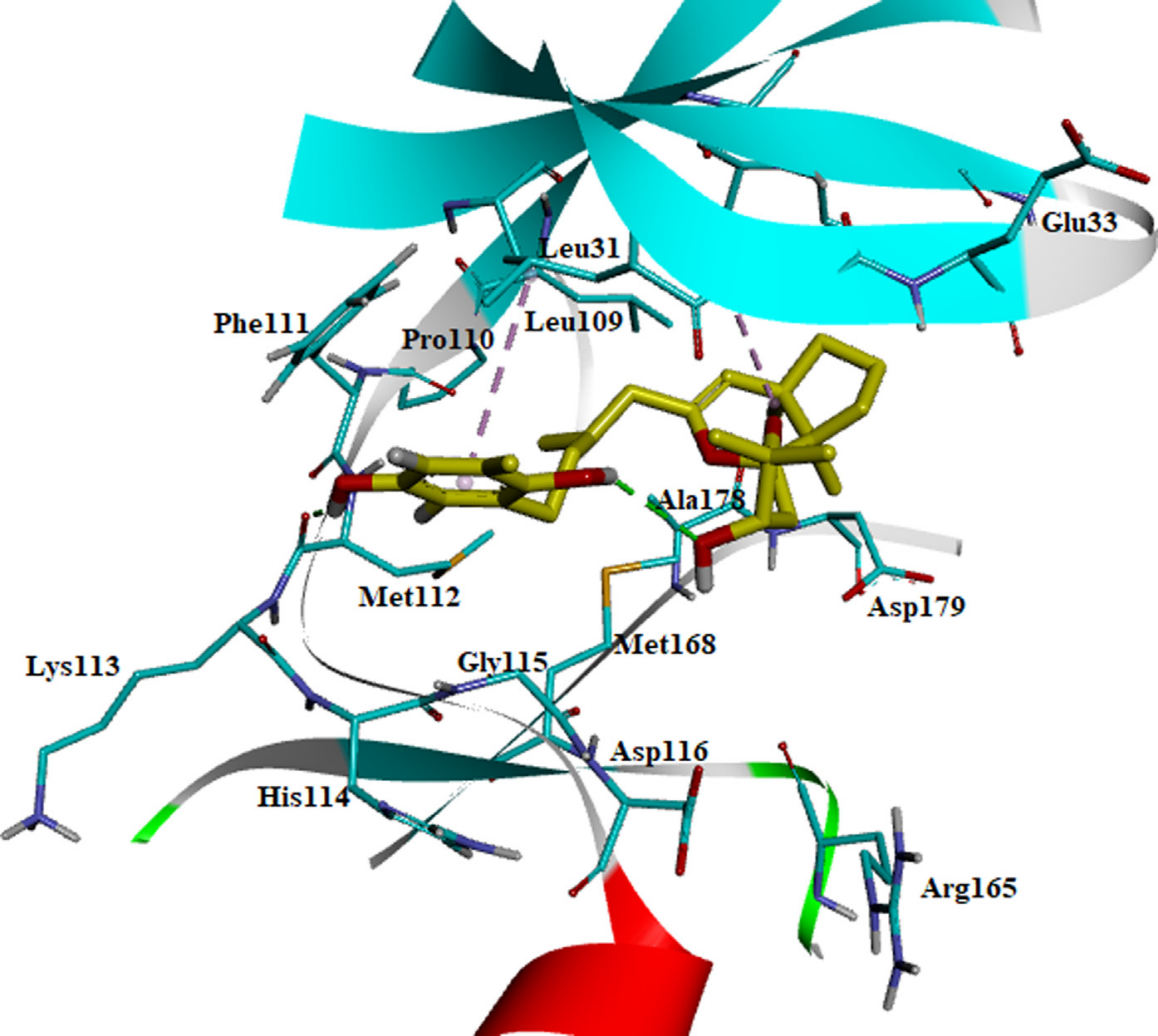
Stabilized average structure of AXL kinase bound to BC005 molecules from molecular dynamics simulation.

Four representative AXL kinase complexes with BC005, BC010, BE005, and GA011 molecules among ten were subjected for the simulations of MD to understand the complexes stability in the water solvent environment. The ‘RMSD’ is the root-mean-square deviation and the ‘RMSF’ is the root mean square fluctuation plots of the complexes from 100 ns of simulations give the stability of the protein complex and the efficiency of the molecule from its interactions with the active site. The AXL protein in complex with the BC005 molecule at 0.45 nm and the last 20 nano seconds the RMSD convergence was observed (Fig. 9). From the trajectory of the complex, the ligand molecule fluctuated at initial simulations was observed and then this ligand stabilized throughout the simulations. The RMSF plot of the BC005 complex showed that the protein stabilized on average at around 0.1 nm fluctuation. From the RMSF plot, two regions of the proteins are crossed at 0.5 nm a fluctuation was observed (Fig. 10). The atom numbers 906–987 represent a loop with Gln95- Pro103 residues connecting two β strands located on the N-terminal domain. The Arg125-Asp128 residues represent the atoms 1204–1243 at loop region have shown high fluctuation. The atom numbers 1891–1987 represents the activation loop of the kinase with Tyr192-Lys199 amino acids. The active site residues are not showing huge fluctuations and the molecules are almost stabilized throughout the MD simulations. The movement of the hinge region residues such as Lys113, His114, and active site residue Met168 involved in the ligand deviation towards the inside of the cavity of the AXL kinase. The ligand retained its hydrogen bond with the hinge region foremost chain NH of Met112 residue. The aromatic ring of the ligand molecule is stabilized by interactions of CH-π with the Leu31 adjacent chain of (Fig. 11).

**Fig. 12:**
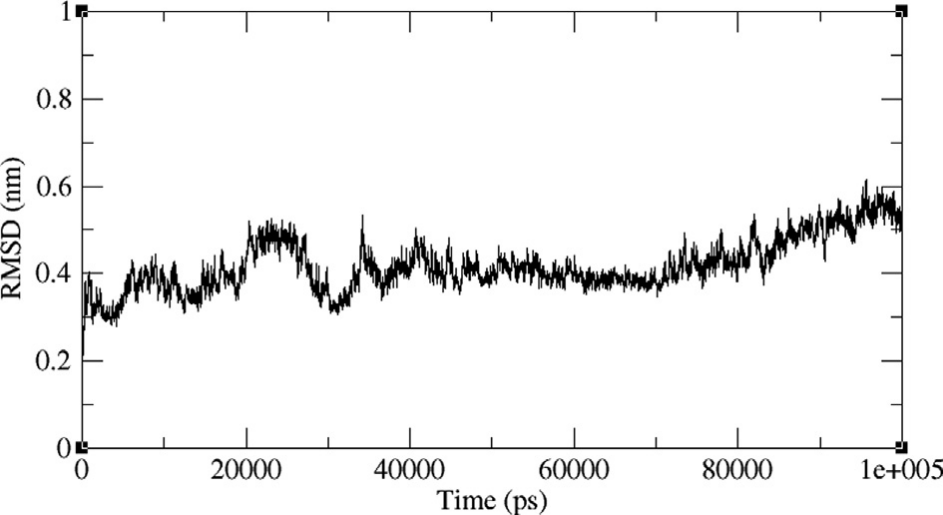
RMSD plot of AXL kinase protein in complex with BC010 molecule.

**Fig. 13:**
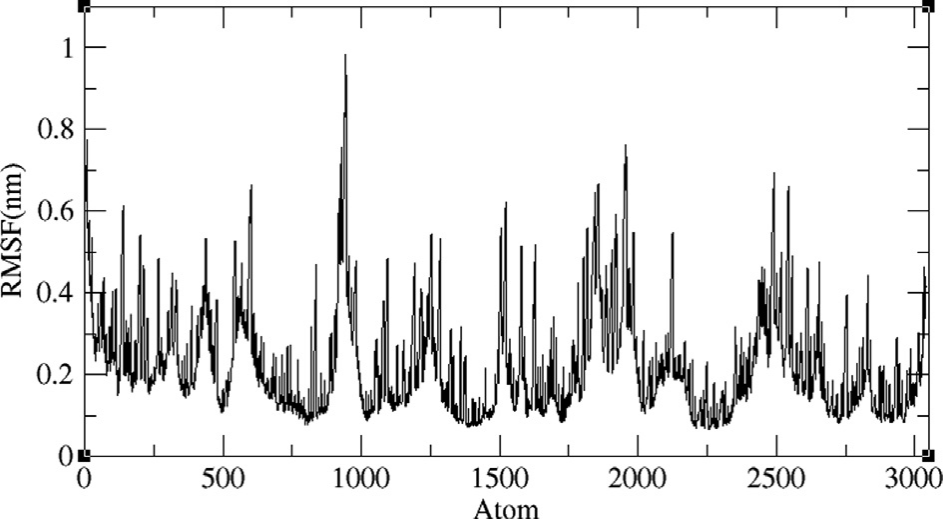
RMSF plot of AXL kinase protein in complex with BC010 molecule.

**Fig. 14:**
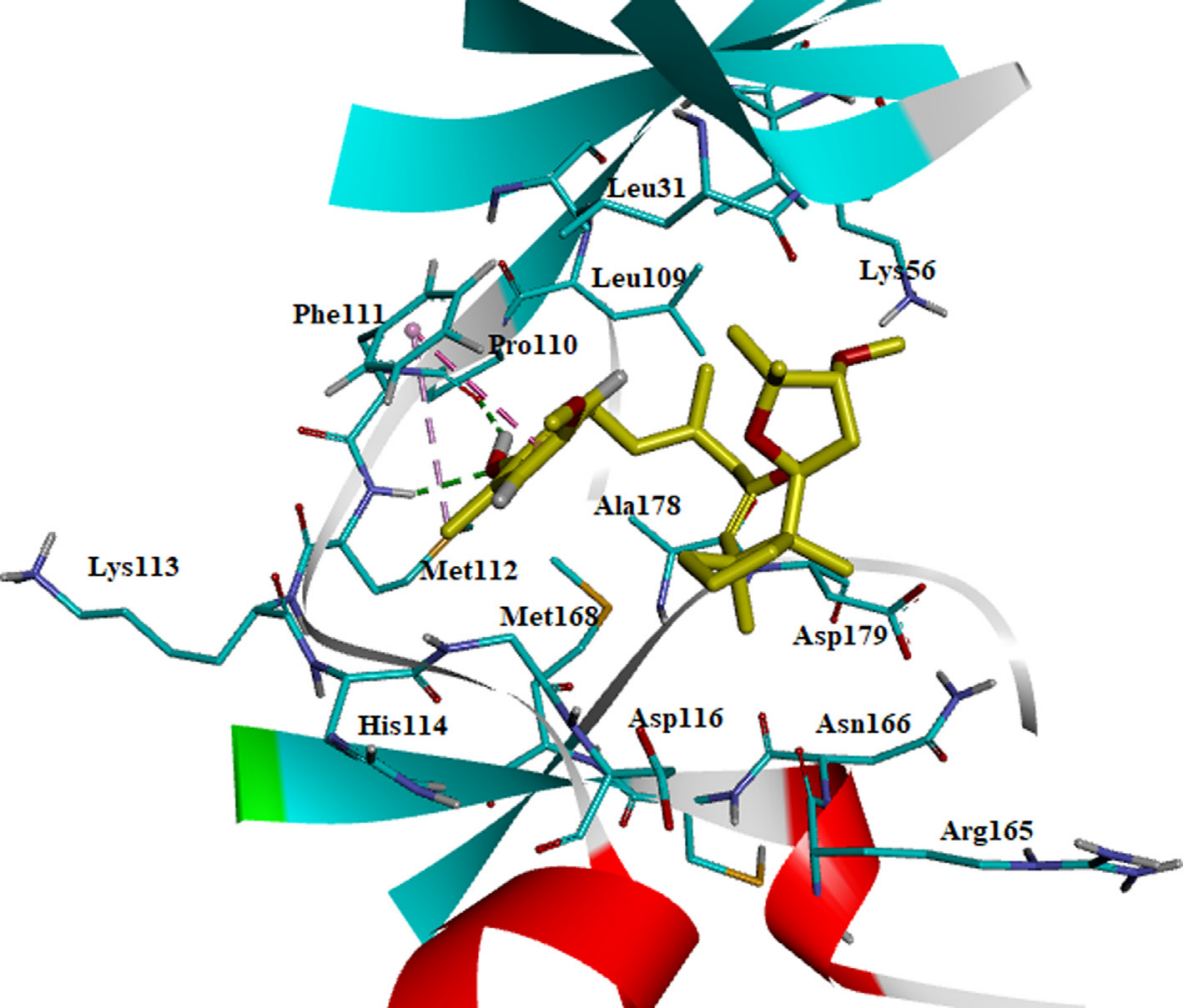
Stabilized average structure of AXL kinase bound to BC010 molecules from molecular dynamics simulation.

The BC010 is molecule complex with AXL kinase is stabilized during the simulations of the MD. The RMSD of the protein is showing the convergence during the last seven nano seconds of the simulations at 0.3 nm (Fig. 12). The RMSF plot of the BC010 complex total is protein stabilized on average of around 0.11 nm fluctuations (Fig. 13). The 0.63 nm fluctuation observed at the atoms 909–958 represents Gly96-Glu100 amino acid residues connecting two β strands located on the N-terminal domain. The atoms of the 1787–1968 region represent Ser183 - Ile197 amino acids at the activation loop is also showing the fluctuation between 0.4 and 0.6 nm. The overall active site was stable and the residues in the active site strongly held the ligand molecule. From the simulation trajectory, very less fluctuation in the ligand molecule was observed. The ligand retained its hydrogen bond with the hinge region main chain NH of Met112 residue and developed an H_2_ bond new with Pro110. The aromatic ring of the ligand molecule is stabilized by π -π interactions with the side chain of Phe111 (Fig. 14).

**Fig. 15:**
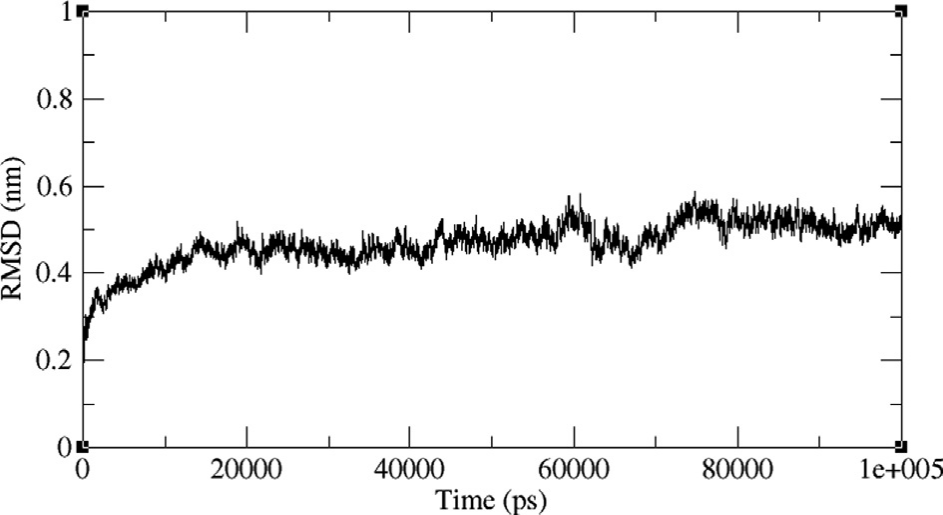
RMSD plot of AXL kinase protein in complex with BE005 molecule.

**Fig. 16:**
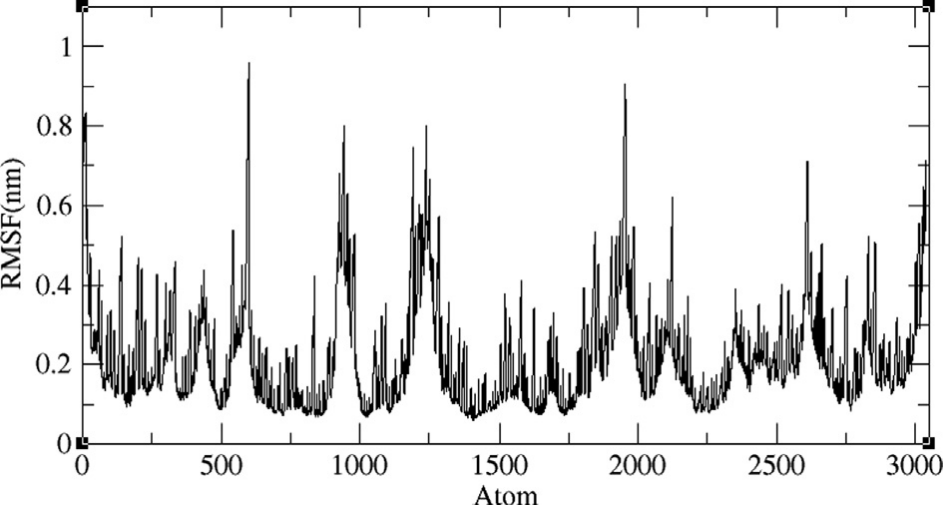
RMSF plot of AXL kinase protein in complex with BE005 molecule.

**Fig. 17:**
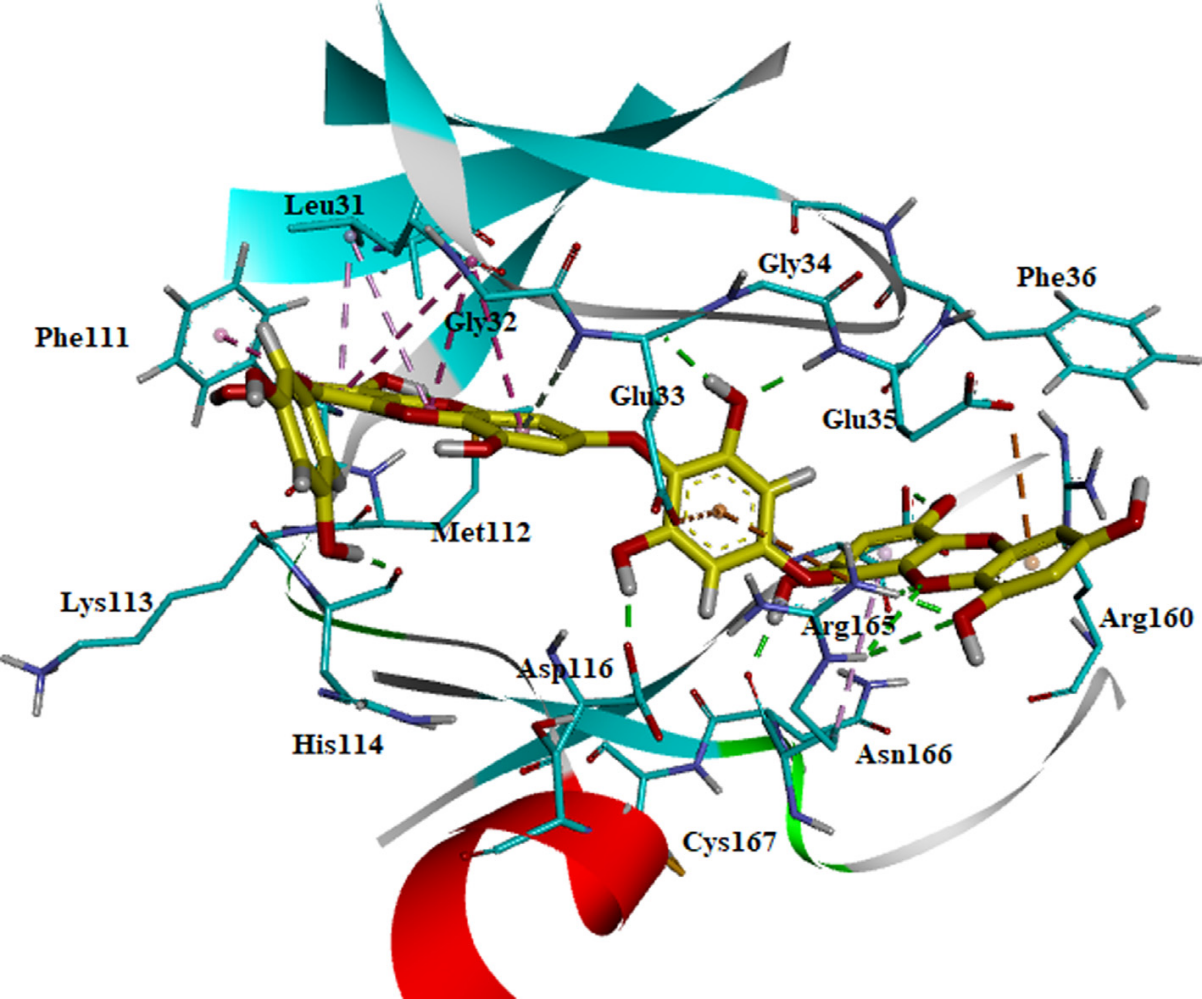
Stabilized average structure of AXL kinase bound to BE005 molecules from molecular dynamics simulation.

The BE005 molecule complex with AXL kinase is stabilized during the MD simulations. The RMSD of the protein is showing the convergence during the last ten nano seconds of the simulations at 0.5 nm (Fig. 15). The RMSF plot of the BE005 complex total is protein stabilized on average of around 0.15 nm fluctuations (Fig. 16). The activation loop and the C-terminal domain loops are showing the RMSF more than 0.5 nm and the higher fluctuation of these loops reached 0.9 nm. The active site of the protein was stable and the binding of the ligand in the active site was retained in a similar location. The molecule is now forming a hydrogen bond with the in the hinge region main chain carbonyl of His114 residue (Fig. 17). The side-chain –OH groups of the ligand also form H_2_ bonds with other resides like focal chain carbonyl oxygen of His114, adjacent chain Asp116 carbonyl oxygen, main-chain NH of Glu35, and side-chain carbonyl groups of Glu33 residues. Ligand molecule oxanthrene is making CH-π interactions with side chains of Leu31, and the other oxanthrene of the ligand is forming three hydrogen bonds with the main chain and adjacent chain nitrogen of Arg165 residue.

**Fig. 18:**
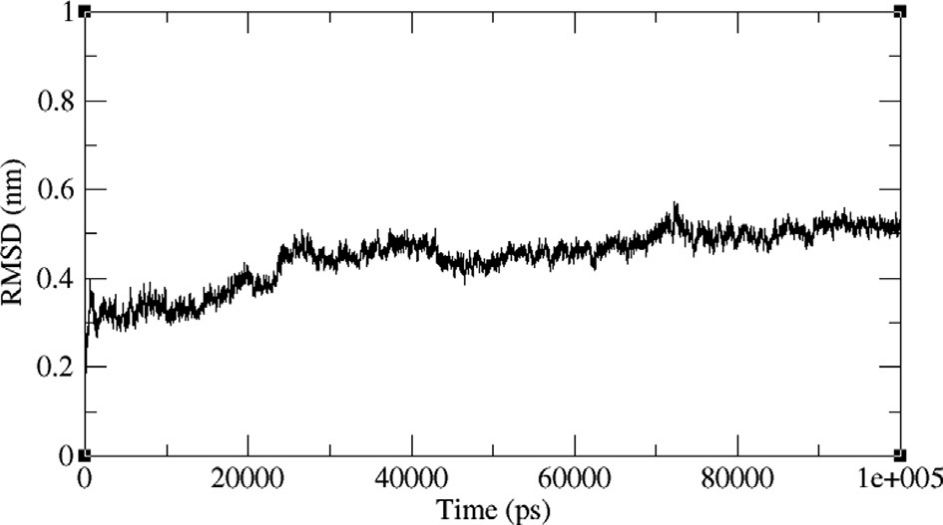
RMSD plot of AXL kinase protein in complex with GA011 molecule.

**Fig. 19:**
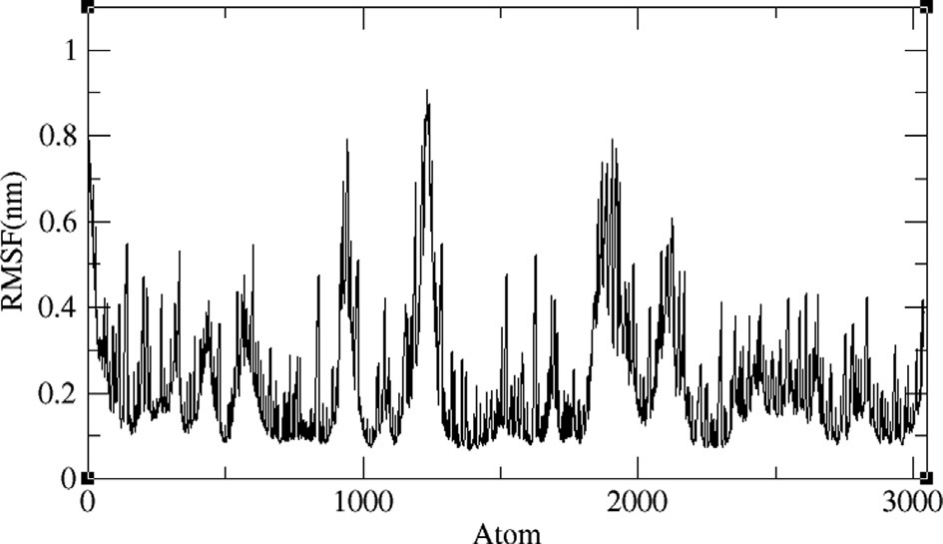
RMSF plot of AXL kinase protein in complex with GA011 molecule.

**Fig. 20:**
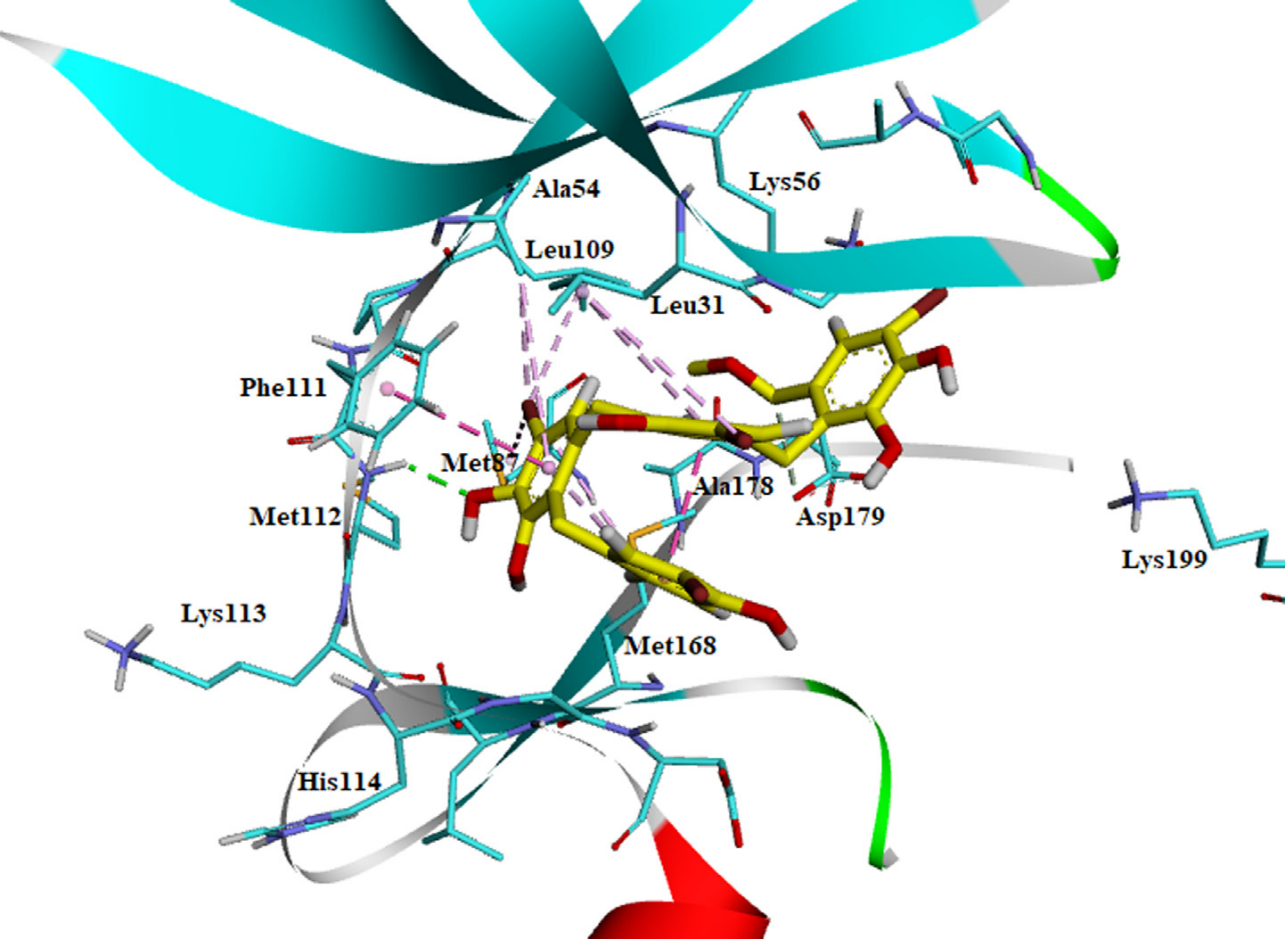
Stabilized average structure of AXL kinase bound to GA011 molecules from molecular dynamics simulation.

The GA011 is molecule complex with AXL kinase is stabilized during the MD simulations. The RMSD of the protein is showing the convergence during the last seven nano seconds of the simulations at 0.5 nm (Fig. 18). The RMSF plot of the GA011 complex total is protein stabilized on average of around 0.15 nm fluctuations (Fig. 19). During the simulations, the two hydroxyl OH groups on Bromo di-hydroxy benzyl ring is establishing a hydrogen bond with the main chain NH and carbonyl oxygen of Met112, residue (Fig. 20). The aromatic benzyl ring is stabilized by π -π interaction with the side chain of Phe111 and CH -π interaction with Ala54 residues. The molecule is stabilized by hydrophobic interactions.

### 4.2 Binding free energy MM-PBSA

The selected complexes MM-PBSA were performed via *g_mmpbsa* tool (Table 1). It uses a MmPbSaStat.py tool to estimate the average binding free energies of the complex is a cumulative total of its components like molecular mechanics, non-polar and polar energies. The free energies binding negative value indicates the strong binding of the molecule into the active site of the protein.

**Table 1:** MM-PBSA binding free energies of the four molecules in complex with AXL kinase are given in kJ/mol.

**Molecule**	**Van der Waals**	**Electrostatic energy**	**Polar solvation energy**	**SASA energy**	**ΔG Free energy of binding**
BC005	−149.27 ± 71.12	−19.89 ± 11.08	80.49 ± 42.60	−14.02 ± 7.45	−102.69 ± 56.01
BC010	−184.59 ± 8.65	−16.58 ± 5.69	83.46 ± 10.08	−17.67 ± 0.85	−135.38 ± 16.67
BE005	−250.49 ± 21.31	−142.24 ± 22.14	276.18 ± 62.29	−25.16 ± 1.30	−141.72 ± 43.63
GA011	−111.99 ± 122.77	−27.30 ± 30.45	78.32 ± 98.151	−11.01 ± 10.93	−71.97 ± 88.91

Two molecules BC010 and BE005 are shown strong binding based on the mmpbsa binding free energies −135.38 kJ/mol and −141.72 kJ/mol respectively. Both the molecules BC010 and BE005 have strong van der Waals energies −184.59 ± 8.65 kJ/mol and −250.49 ± 21.31 kJ/mol, and SASA energies −17.67 ± 0.85 kJ/mol and −25.16 ± 1.30 kJ/mol respectively as free energy components. BC005 molecule is also showing similar energy components and the binding free energy is −103.54 ± 9.66. Due to high polar salvation energy (298. ± 45.37 kJ/mol). The BE005 molecule is having a good number of hydrogen bonds throughout MD simulations and the molecules are showing high binding free energy compared other three molecules. The molecule is having high negative electrostatic (-142.24 ± 22.14 kJ/mol) and positive polar salvation energy (276.18 ± 62.29 kJ/mol) to reduce the total negative value of binding free energy.

**Fig. 21:**
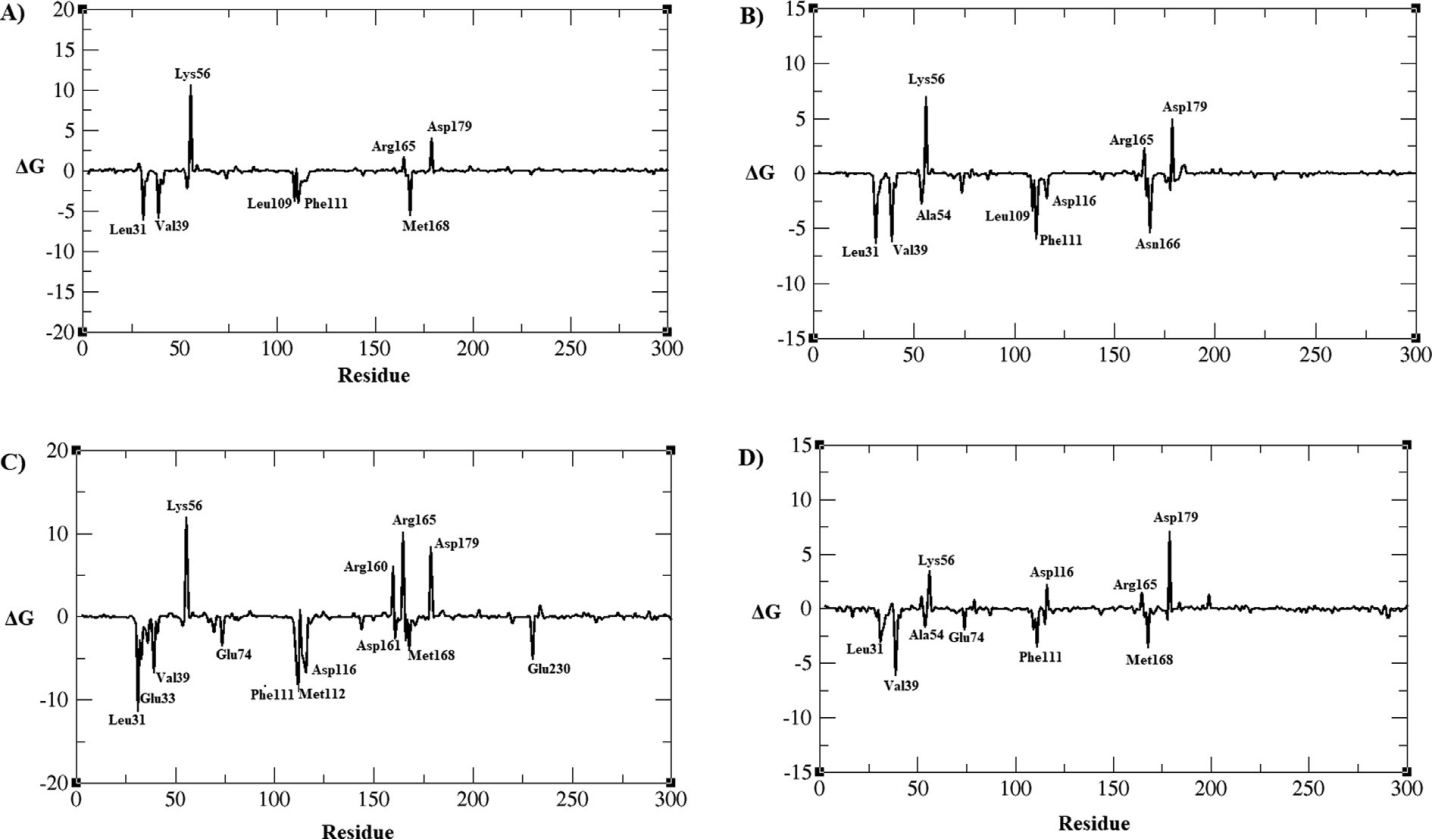
Residues wise free energy decomposition A) BC005 bound AXL kinase B) BC010 bound AXL kinase C) BE005 bound AXL kinase D) GA011 bound AXL kinase and the energies are shown in kJ/mol.

From the free energy decomposition of residues data, the amino acids Leu31, Glu33, Val39, phe111, Met168, leu109, and Asn166 residues are showing strong negative free energies, and the Lys56, Arg165, and Asp179 residues are with positive free energy was observed in all four plots (Fig. 21). All these residues are in the vicinity of the ligand molecule and some of the residues are highly interacting with the molecules.

## 5 Conclusions

Virtual screening of the natural products into the active site of the AXL kinase yields top 8 molecules with a good binding score and orientation. Among eight four molecules are showing strong binding during molecular docking were considered for the MD simulations and free energy calculations. BC010 (−135.38 kJ/mol) and BE005 (−141.72 kJ/mol) are showing good free energy of binding MM-PBSA. The residues active site are also keeping the molecules stale in the active site stable and with low RMSD. The vital residues Leu31, Val39, Glu74, Phe111, Leu109, and Met168 are showing negative binding free energies and also interact in the active site.

## Declaration of Competing Interest

The authors declare that they have no known competing financial interests or personal relationships that could have appeared to influence the work reported in this paper.
